# Favorable cervical cancer mortality-to-incidence ratios of countries with good human development index rankings and high health expenditures

**DOI:** 10.1186/s12905-023-02423-y

**Published:** 2023-05-25

**Authors:** Tzu-Tsen Shen, Cheng-Yu Long, Ming-Ping Wu

**Affiliations:** 1 Division of Urogynecology, Department of Obstetrics and Gynecology, Chi Mei Foundation Hospital, Tainan, Taiwan; 2 Department of Obstetrics and Gynecology, Kaohsiung Medical University Hospital, Kaohsiung Medical University, Kaohsiung, Taiwan; 3 Department of Post-Baccalaureate Medicine, College of Medicine, National Sun Yat-Sen University, Kaohsiung, Taiwan; 4 Department of Obstetrics and Gynecology, Chi Mei Foundation Hospital, 901, Chung Hwa Rd Yung Kang, Tainan, 710 Taiwan

**Keywords:** Cervical cancer, Mortality, Incidence, Mortality-to-incidence ratio, Expenditure

## Abstract

**Background:**

Cervical cancer is highly preventable. The mortality-to-incidence ratio (MIR) is a marker that reflects the available screening interventions and clinical outcomes of cancer treatments. The association between the MIR for cervical cancer and cancer screening disparities among countries is an interesting issue but rarely investigated. The present study sought to understand the association between the cervical cancer MIR and the Human Development Index (HDI).

**Methods:**

Cancer incidence and mortality rates were obtained from the GLOBOCAN database. The MIR was defined as the ratio of the crude mortality rate to the incidence rate. We used linear regression to analyze the correlation of MIRs with the HDI and current health expenditure (CHE) in 61 countries selected based on data quality.

**Results:**

The results showed lower incidence and mortality rates and MIRs in more developed regions. In terms of regional categories, Africa had the highest incidence and mortality rates and MIRs. The incidence and mortality rates and MIRs were lowest in North America. Furthermore, favorable MIRs were correlated with a good HDI and high CHE as a percentage of gross domestic product (CHE/GDP) (both p < 0.0001).

**Conclusions:**

The MIR variation for cervical cancer is associated with the ranking of the health system and health expenditure, which further supports the role of cancer screening and treatment disparities in clinical outcomes. The promotion of cancer screening programs can reduce the cervical cancer global incidence and mortality rates and MIRs.

## Background

Cervical cancer is the third most common gynecologic cancer in women, 90% of which occur in low- and middle-income countries [1]. In 2020, there were estimated 604,000 new cases of cervical cancer and estimated 342,000 cervical cancer deaths worldwide, among them about one-fifth occurred in Africa (117,000 cases and 77,000 deaths) [2]. However, cervical cancer is highly preventable and treatable [3]. Investment in screening programs may decrease the incidence rate and improve clinical outcomes.

Global incidence and mortality rates depend on the promotion of cancer screening programs, which are more likely to be available in developed regions. In terms of cervical cancer, favorable socioeconomic conditions enable the screening of precancerous lesions or the detection of early-stage cancer by Pap smear and human papillomavirus (HPV) testing. Patients with early cancer detection can be treated with more simplified treatment modalities, then avert complex cancer treatments, such as radical hysterectomy and concurrent chemoradiotherapy, which impact the life quality and mortality rate [4, 5]. The World Health Organization (WHO) approved a global strategy, which aimed to eliminate cervical cancer among developing countries in 2020. The elimination initiative suggested a three-pillar approach and hope to achieve by 2030. The 2030 targets include: 90% of eligible girls fully vaccinated against HPV by 15 years of age; 70% of eligible women screened with a high-precision test at 35 years and at 45 years; and 90% of women identified with cervical disease receive treatment and care. The Director General of the WHO requested countries to forge partnerships with all actors for coordinated action [6].

Using Taiwan as an example, the National Health Insurance (NHI) has paid for the Pap smear screening for women over 30 since 1995, which could explain the yearly decrease in morbidity after 1998. In terms of the period effect, the mortality trend decreased two-fold from 1996 to 2010 [7].

HPV vaccination is an another way to decrease the cancer incidence rate [8]. The HPV vaccine is recommended to reduce HPV infection, especially for girls between the ages of 9 to 13. Some European countries (e.g., Sweden) have implemented the national HPV immunization program, which has resulted in an 80% coverage rate and a reduced risk of cervical cancer [9]. In Taiwan, the quadrivalent and bivalent HPV vaccines have been available since 2006 and 2008, respectively. Due to government support in providing free HPV vaccination to young girls (9–15 years old) since 2018, a future decline in the prevalence and incidence of cervical cancer can be expected [10]. Whether further devotions of financial expenditures have impacts on disease outcome deserves our attention.

The mortality-to-incidence ratio (MIR) is defined as the ratio of the crude mortality rate to the incidence rate. It is used as a marker to reflect the available screening interventions and clinical outcomes of cancer treatments. Previous studies found that development and health expenditure were related to the MIRs for prostate and colon cancer [11, 12]. Therefore, we hypothesized that the MIR also impacts cervical cancer in countries with varying healthcare systems. The purpose of the present study aimed to evaluate the impacts of different economic statuses on the MIR for cervical cancer, which may further supports the role of cancer screening and treatment disparities in clinical outcomes.

## Methods

### Data acquisition

Data relating to cancer incidence and mortality were obtained from the 2018 GLOBOCAN database, a public database comprising data on 185 countries and maintained by the International Agency for Research on Cancer (https://www.iarc.fr/). The MIR was defined as the ratio of the crude rate of mortality to the incidence. It is a novel measure that can be used to evaluate cancer mortality in relation to incidence as a proxy for survival [12]. The crude rate and age-standardized rate (ASR) are multiplied by 100,000 (cases per 100,000 inhabitants). The Human Development Index (HDI) is a ranking system based on indicators such as life expectancy, education, and per capita income. The data were obtained from the Human Development Reports database of the United Nations Development Programme (http://hdr.undp.org/). The Current health expenditure per capital (CHE per capita) and CHE as a percentage of gross domestic product (CHE/GDP) were obtained from the WHO statistics report (https://www.who.int/; World Health Statistics 2018).

The exclusion criteria for the selection of countries for this study included missing data in the WHO statistics (n = 12), missing HDI data (n = 2), or cases where there was low data availability (n = 110). Consequently, a total of 61 countries were included in the final analysis.

### Statistical analyses

The association between the MIR and variants were estimated via linear regression. R^2^ changes and analysis of variance were determined using the SPSS statistical software, version 15.0 (SPSS, Inc., Chicago, IL). P values < 0.05 were considered statistically significant. Scatter plots were produced using Microsoft Excel 2010.

## Results

### Incidence and mortality rate of cervical cancers by region

The incidence and mortality, crude rates, age-standardized rates (ASRs), and MIRs from different regions of the world are summarized in Table 1. In terms of the WHO’s regional categories, Africa had the highest incidence and mortality based on ASR (27.4 and 19.7, respectively), whereas North America had the lowest (6.4 and 1.9, respectively). Compared to less developed regions, the more developed regions had a much lower MIR (0.36 for more developed vs. 0.68 for less developed).

**Table 1:** Summary of the number, CR, ASR of incidence and mortality, and MIR for cervical cancer according to region

	New cases			Deaths			MIR
Region	Number	CR	ASR	Number	CR	ASR	
Africa	118,329	18.4	27.4	80,755	12.6	19.7	0.68
Asia	311,021	14.1	11.9	162,485	7.4	6.1	0.52
Europe	59,297	15.9	11.2	23,722	6.4	3.7	0.40
Latin America and the Caribbean	54,378	16.7	14.5	26,533	8.1	6.9	0.49
North America	15,048	8.4	6.4	5,348	3.0	1.9	0.36
Oceania	2,408	11.9	10.2	1,211	6.0	4.7	0.50

CR: crude rate; ASR: age-standardized rate; MIR: mortality-to-incidence ratio

### Incidence and mortality of cervical cancers by country

The CHE and MIRs for cervical cancer in the 61 representative countries which selected based on their HDI ranking are summarized in Table 2. Among the 61 countries, South Africa had the highest incidence of crude rates and ASR. Three countries had high mortality ASR values greater than 18: South Africa (18.6), Fiji (19.1), and Jamaica (19.7).

**Table 2:** Summary of current health expenditure, cancer incidence and mortality, and MIR for cervical cancer according to their HDI ranking (N = 61)

		CHE		Incidence			Mortality			
Country	HDI	Per Capita	% of GDP	Number	CR	ASR	Number	CR	ASR	MIR
Egypt	0.7	157	4.2	932	1.9	2.2	594	1.2	1.4	0.63
South Africa	0.705	471	8.2	12,779	43.9	43	5407	18.6	18.6	0.42
Philippines	0.712	127	4.4	7116	13.5	14.7	3980	7.5	8.5	0.56
Fiji	0.724	175	3.6	123	27.4	25.6	92	20.5	19.1	0.75
Jamaica	0.726	294	5.9	470	32.8	28.1	338	23.6	19.7	0.72
Ukraine	0.75	125	6.1	5677	24.5	17	2426	10.5	6.6	0.43
Ecuador	0.758	530	8.5	1521	18.2	17.3	750	9	8.4	0.49
Brazil	0.761	780	8.9	15,841	14.9	12	7664	7.2	5.7	0.48
Colombia	0.761	374	6.2	3731	15	12.5	1659	6.7	5.5	0.45
Thailand	0.765	217	3.8	8429	24.1	16.1	4873	13.9	8.9	0.58
Cuba	0.778	826	10.9	1187	21.2	14.5	556	9.9	5.9	0.47
Costa Rica	0.794	929	8.1	335	13.7	11	170	6.9	5.2	0.5
Mauritius	0.796	506	5.5	114	18	12.1	53	8.4	5.2	0.47
Serbia	0.799	491	9.4	1313	30	20.4	522	11.9	6.9	0.4
Trinidad and Tobago	0.799	1146	6	138	20	15.2	91	13.2	9.1	0.66
Malaysia	0.804	386	4	1667	10.8	10.5	918	5.9	5.8	0.55
Kuwait	0.808	1169	4	59	3.3	3.4	31	1.7	2.2	0.52
Uruguay	0.808	1281	9.2	274	15.8	12.4	147	8.5	5.9	0.54
Bulgaria	0.816	572	8.2	1062	30.1	20.3	453	12.9	7.3	0.43
Belarus	0.817	352	6.1	958	19.4	13.3	300	6.1	3.8	0.31
Russian Federation	0.824	524	5.6	17,922	23.8	17	7321	9.7	6.2	0.41
Argentina	0.83	998	6.8	4381	19.6	16.7	2098	9.4	7.6	0.48
Oman	0.834	636	3.8	76	4.7	6.2	40	2.5	3.8	0.53
Croatia	0.837	852	7.4	251	12	7.8	151	7.2	3.5	0.6
Bahrain	0.838	1190	5.2	19	3.3	3.8	12	2.1	2.7	0.64
Chile	0.847	1102	8.1	1449	16.1	12	638	7.1	4.8	0.44
Qatar	0.848	2030	3.1	19	2.8	4	12	1.8	3.2	0.64
Portugal	0.85	1722	9	697	13.4	8.8	289	5.5	2.7	0.41
Latvia	0.854	784	5.8	334	33.2	25.1	121	12	6.4	0.36
Slovakia	0.857	1108	6.9	677	24.7	16.6	265	9.7	5.6	0.39
Lithuania	0.869	923	6.5	415	27.8	18.9	189	12.6	7	0.45
Poland	0.872	797	6.3	3043	15.9	9.3	1756	9.2	4.7	0.58
Cyprus	0.873	1563	6.8	44	7.5	5.7	15	2.6	1.3	0.35
Estonia	0.882	1112	6.5	220	32.9	22.4	54	8.1	4.2	0.25
Italy	0.883	2700	9	2980	10.3	7.1	860	3	1.5	0.29
Malta	0.885	2304	9.6	11	5.2	3.5	6	2.9	1.4	0.56
Czechia	0.891	1284	7.3	781	14.9	9.9	391	7.4	3.8	0.5
France	0.891	4026	11.1	2870	9.1	6.7	1209	3.8	2.2	0.42
Spain	0.893	2354	9.2	1833	8.1	5.1	702	3.1	1.6	0.38
Slovenia	0.902	1772	8.5	107	10.6	7.1	56	5.6	2.7	0.53
Israel	0.906	2756	7.4	231	5.5	4.8	115	2.8	2.1	0.51
South Korea	0.906	2013	7.4	3188	12.7	8.3	868	3.5	1.8	0.28
Luxembourg	0.909	6236	6	23	8.1	5.5	9	3.2	1.9	0.4
Austria	0.914	4536	10.3	364	8.5	5.4	138	3.2	1.6	0.38
Japan	0.915	3733	10.9	12,519	20.4	14.7	3245	5.3	2.6	0.26
Belgium	0.919	4228	10.5	608	10.9	7.7	193	3.4	1.9	0.31
UK	0.92	4356	9.9	3314	10.2	8.4	897	2.8	1.6	0.27
US	0.92	9536	16.8	13,659	8.5	6.5	4833	3	1.9	0.35
New Zealand	0.921	3554	9.3	182	7.7	6	64	2.7	1.7	0.35
Canada	0.922	4508	10.4	1386	7.7	5.7	515	2.8	1.7	0.36
Finland	0.925	4005	9.4	168	6.2	4.7	51	1.9	0.9	0.31
Denmark	0.93	5497	10.3	399	14.2	10.9	106	3.8	1.8	0.27
Netherlands	0.934	4746	10.7	637	7.7	5.6	210	2.5	1.4	0.32
Singapore	0.935	2280	4.3	374	13	7.1	184	6.4	3.6	0.49
Sweden	0.937	5600	11	530	11	8.9	180	3.7	1.9	0.34
Australia	0.938	4934	9.4	903	7.5	6	305	2.5	1.6	0.33
Iceland	0.938	4375	8.6	14	8.5	7.5	3	1.8	1.4	0.21
Germany	0.939	4592	11.2	4442	11.1	7.5	1754	4.4	2.1	0.4
Ireland	0.942	4757	7.8	335	14.1	11	101	4.3	2.8	0.3
Switzerland	0.946	9818	12.1	241	5.8	3.8	83	2	1	0.34
Norway	0.954	7464	10	349	13.6	10.7	72	2.8	1.6	0.21

ASR: age-standardized rate; CHE: Current Health Expenditure; CR: crude rate; MIR: mortality-to-incidence ratio; HDI: health development index; UK: United Kingdom; US: United States of America

Considering the United States as the representative of high-income Western countries, the ASRs relating to both the incidence and mortality of cervical cancer were only 6.5 and 1.9, respectively. Using Japan as the representative of high-income countries in the Asia, and with data most similar to Taiwan, the ASRs relating to the incidence and mortality of cervical cancer were 14.7 and 2.6, respectively.

**Fig. 1:**
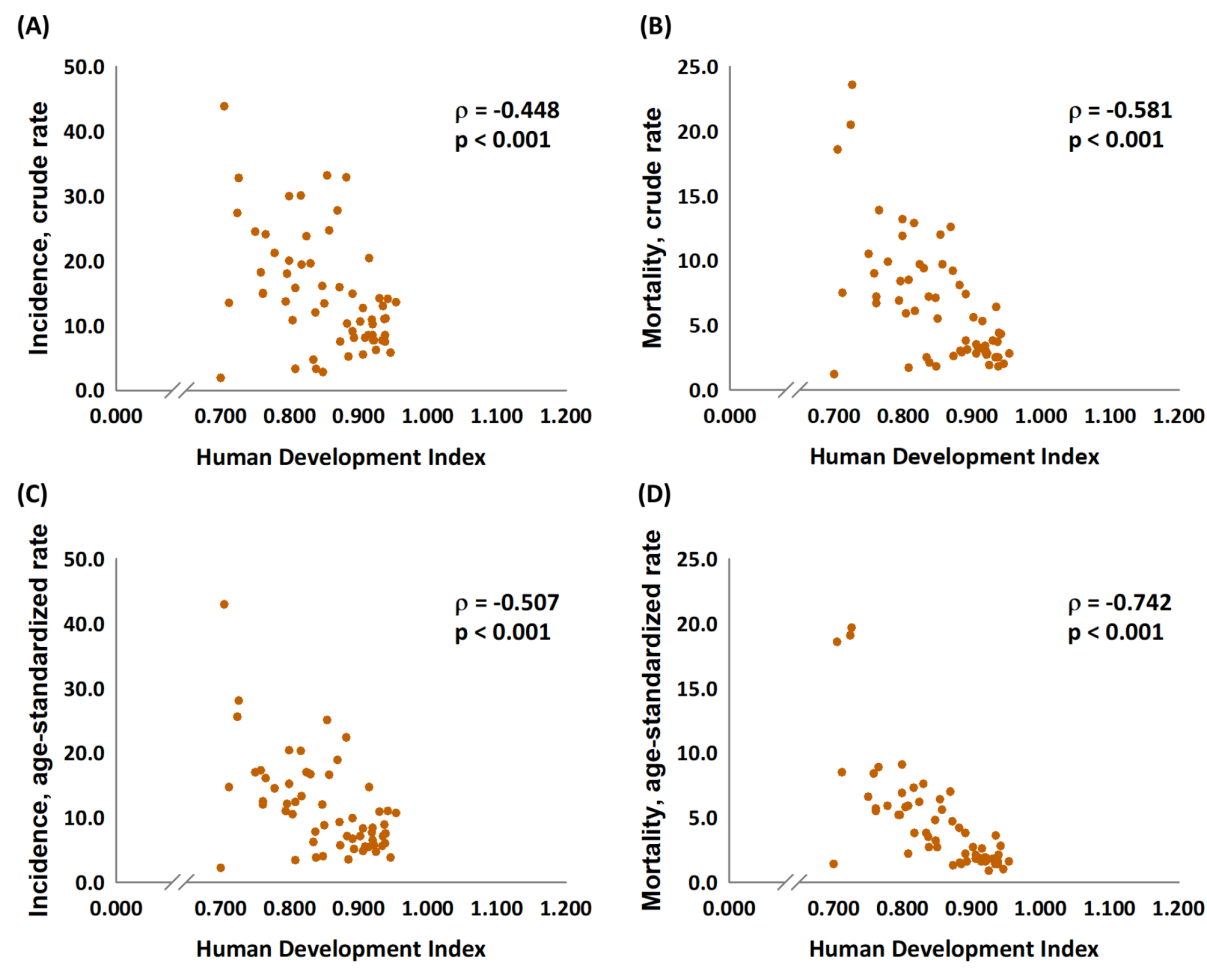
The association between the HDI and the incidence and mortality in crude rates (**A** and **B**); The association between the HDI and the incidence and mortality in age-standardized rates (**C** and **D**) of cervical cancer

**Fig. 2:**
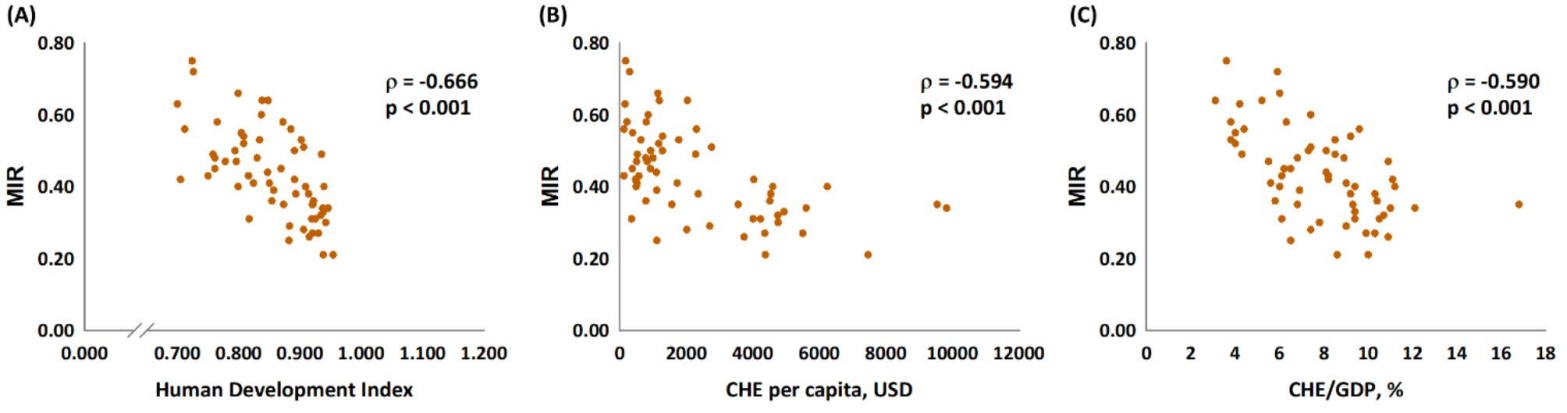
The (**A**) HDI, (**B**) current health expenditure per capita, and (**C**) current health expenditure as a percentage of GDP are significantly associated with the MIR for cervical cancer

As expected, the HDI was significantly associated with the crude rates and ASRs relating to incidence and mortality (p < 0.001, Fig. 1). The favorable MIRs for the 61 countries were significantly associated with a good HDI, a high CHE per capita, and a high CHE/GDP (p < 0.001, Fig. 2).

## Discussion

Cervical cancer is a common cancer worldwide, especially in developing countries, which constitutes a significant health threat and economic burden on healthcare systems [2]. Previous systematic reviews, meta-analyses, and observational studies have consistently showed that screening program can decrease the incidence of cervical cancer [13]. One study from the Setif Cancer Registry, Algeria (1986–2010), documented decreased cervical cancer incidence rates in the period 1986–2010 (annual percentage change: -4.2%), which was attributed to opportunistic cytology screening [14]. In a randomized trial of over 130,000 patients in rural India, a single lifetime screen with HPV testing reduced cervical cancer mortality by 50%, as compared with no screening (12.7 vs. 25.8 per 100,000 person-years, hazard ratio 0.52, 95% confidence interval 0.33–0.83) [15]. As cervical cancer is one of the most preventable and (if diagnosed early) treatable forms of cancer, it can be regarded as a global health problem.

To our knowledge, this is the first study to investigate the associations among the MIR for cervical cancer and the HDI, CHE. Our study indicated a significant correlation between the HDI and the MIR for cervical cancer in different countries. More support of healthcare expenditure, either through personal funding or country-based programs, led to lower MIRs for cervical cancer. This reflects the importance of the availability of early cancer screening, HPV vaccination programs, and advanced cancer treatments (e.g., surgery, concurrent chemoradiotherapy) in countries with better healthcare rankings.

Although Pap smear screening offers the opportunity for early diagnosis of cervical cancer, many women were diagnosed as invasive cervical cancer cases. Most of them had never been screened or participated in routine screening, even in countries with well-organized screening programs based on cytology and good coverage [16]. In Asian countries (e.g., Taiwan), culture-specific barriers to Pap testing led to a significantly lower coverage among elderly women. Chou et al. explained the reasons for Pap smears avoidance among Taiwanese women, which included fear of discomfort or pain, shyness, lack of medical knowledge, lack of a sense of urgency, busyness, loss of confidence in Pap smear screening, the feeling that it is not possible to get cervical cancer, and not being able to face bad news [17]. In our study, Japan had the higher incidence and mortality of ASR and Asia countries had higher MIR, as compared to Western countries with similar economies (Table 1, 2). One option to solve the problem of low Pap screening percentage is HPV-based screening, which allows for high sensitivity rates over cytology. For Pap smear under-users, HPV-based screening offers the possibility of self-sampling and makes possible longer screening intervals in women with negative screening results. It is a way to improve under-screening by self-sampling with sample kits offered in communities or mailed directly to homes. Based on an updated meta-analysis by Maver et al., the diagnostic accuracy of PCR-based high-risk HPV assays were equally sensitive for underlying CIN2 + or CIN3 + on self-samples versus clinician-collected samples. Some European countries have implemented HPV-based screening since July 2019 [18]. Countries with low Pap smear screening rates may consider the transition to HPV-based cervical cancer screening in the future to improve their MIR.

Different cancer types have different MIR patterns among countries with “different civilization”. We compared cervical cancer to other cancers, for example, lung cancer, colorectal cancer, prostate cancer, liver cancer, gastric cancer, and pancreas cancer. Cervical cancer, as well as colorectal cancer, showed lower incidence and mortality rates and lower MIRs in high-income countries [12]. Well screening guidelines may explain the lower MIRs.

On the contrary, gastric cancer, lung cancer, and prostate cancer had higher incidence and mortality rates but lower MIR values in high-income countries compared to low-income countries. These cancers can be divided into “diseases of civilization” [19]. The high incidence and mortality rates might be associated with the Western diet and more sedentary lifestyle, while early detection methods and early-stage cancer with immediate treatment resulted in low MIRs in higher developed countries [11, 12, 20–23].

Another pattern of association occurred in pancreatic cancer, which had high incidence and mortality rates in developed countries. Meanwhile, its MIR did not correlate with healthcare disparities among countries [22]. This could be because pancreatic cancer is a highly lethal disease and most cases are usually detected in the advanced stages because of no efficient screening methods [1]. Therefore, more healthcare expenditure, either through personal funding or country-based programs, cannot improve disease outcome in this disease pattern.

To our knowledge, this is the first study to address the association among the MIRs for cervical cancer and the HDI, CHE. This can potentially guide government to manage health expenditure among their healthcare systems. Our study showed lower incidence and mortality rates for cervical cancer in countries characterized by a better HDI. The MIRs of different countries were also negatively correlated with the HDIs. The MIR is therefore a potentially useful parameter for monitoring the screening and healthcare treatment status of cervical cancer. Therefore, we need to integrate current interventions into existing health plans to reduce the future burden from cancer.

There were some limitations of our study which need to be taken into account. First, no detail clinical information was analyzed, e.g. cervical cancer stage and screening program. Second, many countries were excluded due to low data availability, with only 61 countries recruited for the final analyses.

In this study, we demonstrated that the MIR for cervical cancer is associated with healthcare disparities. As observed better outcome after the implementation of Pap smear screening tests, we are expecting to see further and better results for HPV vaccination and HPV-based cervical cancer screening. The promotion of highly effective cancer screening programs is a feasible option in Taiwan and other countries. It can improve under-screening problem and reduce the global incidence and mortality rates as well as the MIRs for cervical cancer.

## Data Availability

All data generated or analyzed during this study are included in this published article. All the data used in this study were obtained from the global statistics of GLOBOCAN (https://www.iarc.fr/), the Human Development Reports database of the United Nations Development Programme (http://hdr.undp.org/), and WHO statistics reports (https://www.who.int/; World Health Statistics 2018).

## Abbreviations

HDI: Human Development Index
CHE per capita: Current health expenditure per capital
CHE/GDP: CHE as a percentage of gross domestic product
ASRs: Age-standardized rates
